# Supplementary material for: Asynchronous magnetic resonance elastography: Shear wave speed reconstruction using noise correlation of incoherent waves
Source: Magn Reson Med. 2022 Oct 27;89(3):990–1001. doi: 10.1002/mrm.29502 (PMC9792433; doi:10.1002/mrm.29502)
Supplement: Supplementary file 1 — DATA S1 MATLAB code used to generate the numerical simulations of Figure 1 [file MRM-89-990-s002.zip › k-Wave/helpfiles/grid2cart.html]

grid2cart :: Functions (k-Wave)


# grid2cart

Return the Cartesian coordinates of the non-zero points of a binary grid.

## Syntax

```
[cart_data, order_index] = grid2cart(kgrid, grid_data)
```

## Description

`grid2cart` returns the set of Cartesian coordinates corresponding to the non-zero elements in the binary matrix `grid_data`, in the
coordinate framework defined in `kgrid`.

## Inputs

|  |  |
| --- | --- |
| `kgrid` | k-Wave grid object returned by `kWaveGrid` |
| `grid_data` | binary grid with the same dimensions as the k-Wave grid `kgrid` |

## Outputs

|  |  |
| --- | --- |
| `cart_data` | 1 x N, 2 x N, or 3 x N (for 1, 2, and 3 dimensions) array of Cartesian sensor points |
| `order_index` | the order that the Cartesian points appear in `grid_data` according to MATLAB's standard column-wise linear matrix index ordering |

## See Also

`cart2grid`, `interpCartData`, `kWaveGrid`
